# Supplementary material for: Tuning defects in oxides at room temperature by lithium reduction
Source: Nat Commun. 2018 Apr 3;9:1302. doi: 10.1038/s41467-018-03765-0 (PMC5882908; doi:10.1038/s41467-018-03765-0)
Supplement: Supplementary file 1 — Supplementary Information [file 41467_2018_3765_MOESM1_ESM.pdf]

Supplementary Information for

Tune Defects in Oxides at Room-temperature by Lithium Reduction

Gang Ou, Yushuai Xu, Bo Wen, Rui Lin, Binghui Ge, Yan Tang, Yuwei Liang, Cheng Yang, Kai Huang, Di Zu, Rong Yu, Wenxing Chen, Jun Li, Hui Wu, Li-Min Liu & Yadong Li

Correspondence to Hui Wu (email: huiwu@tsinghua.edu.cn) or to Li-Min Liu (email: liminliu@buaa.edu.cn) or to Yadong Li (email: ydli@tsinghua.edu.cn)

Supplementary Figures

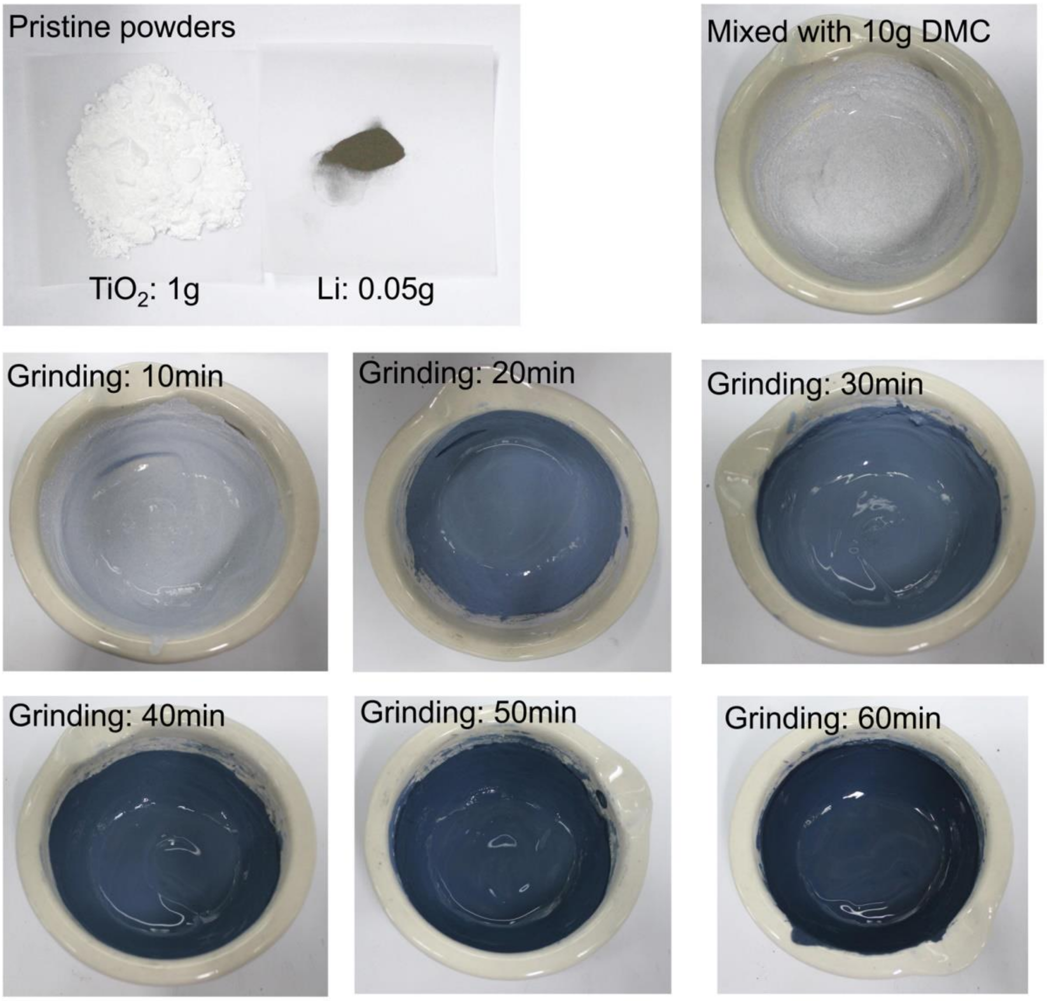

Supplementary Figure 1 | Photograph of detailed process for the preparation of defective TiO_2 nanopowders reduced by 5 wt% Li.

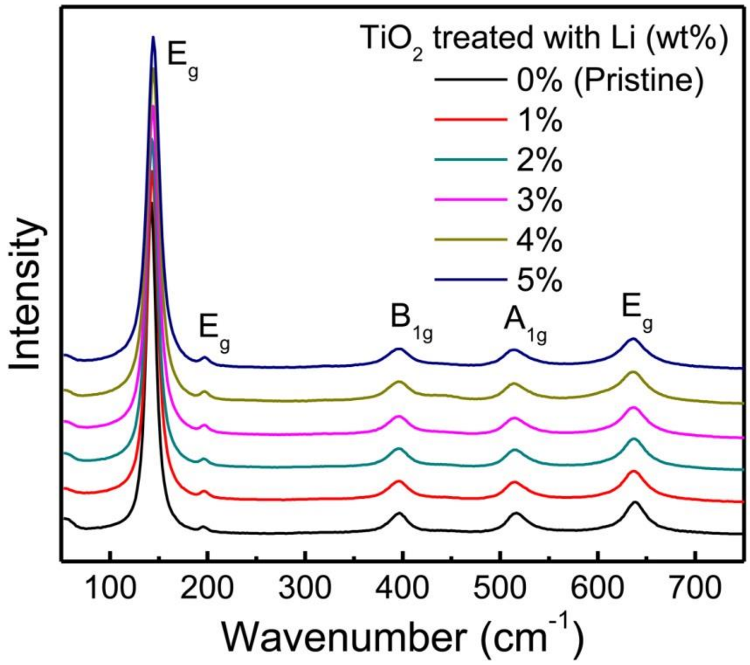

**Supplementary Figure 2 | ** Raman spectra of pristine and lithium reduced TiO_2 nanoparticles. All of the TiO_2 nanoparticles demonstrate almost the same band structure.

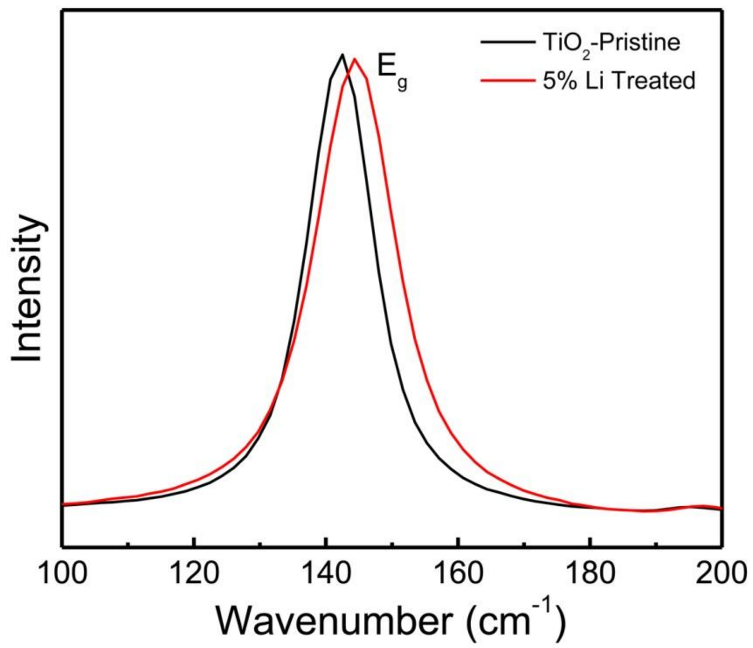

Supplementary Figure 3 | Enlarged Raman spectra of pristine TiO_2 and 5% Li treated TiO_2 nanoparticles. The peak became wider after lithium reduction, suggesting the degree of lattice disorder increased.

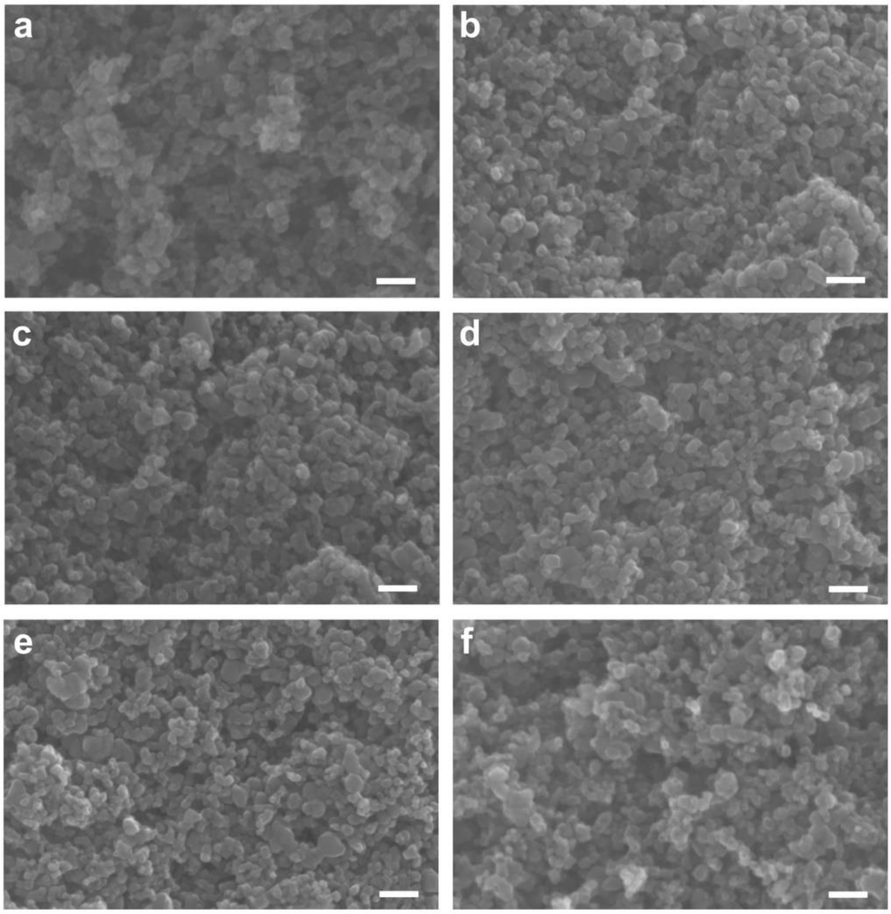

Supplementary Figure 4 | SEM micrographs of pristine and lithium reduced TiO_2 nanoparticles. **a**, Pristine TiO_2. **b**, 1% Li treated. **c**, 2% Li treated. **d**, 3% Li treated. **e**, 4% Li treated. **f**, 5% Li treated. The particle size of TiO_2 nanoparticles keeps almost the same before and after lithium reduction, indicating grain growth did not occurred in the grinding process. Scale bars, 100 nm.

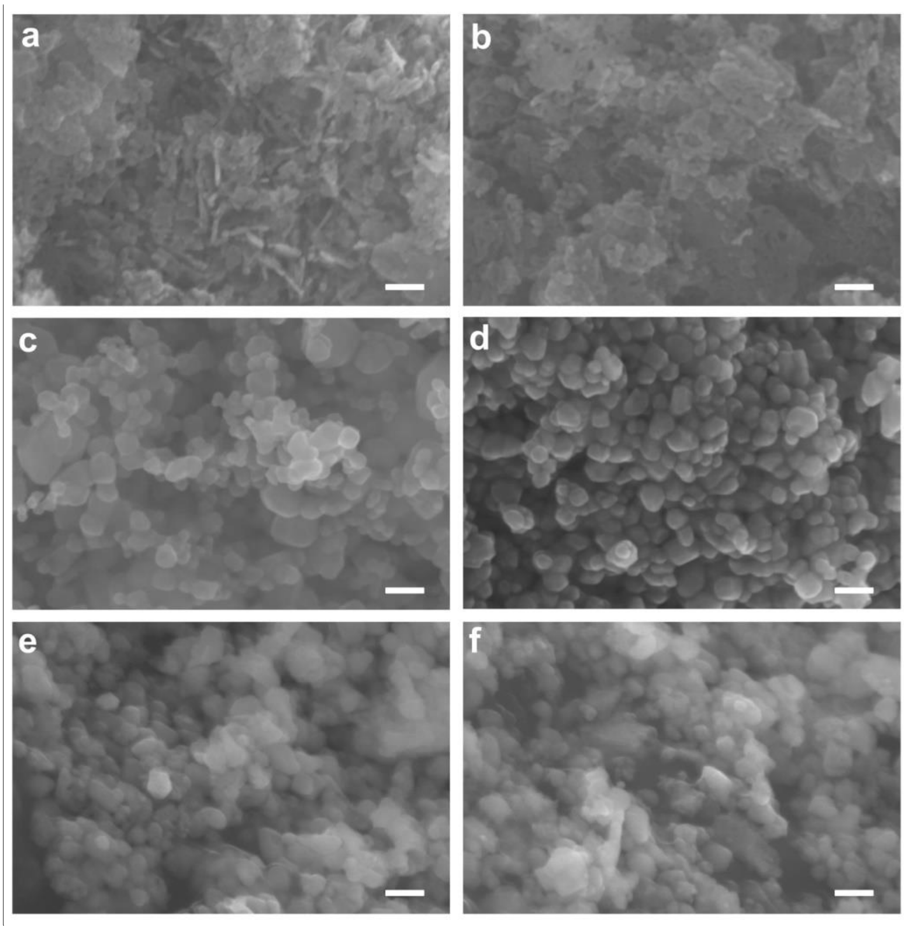

Supplementary Figure 5 | SEM micrographs of pristine and lithium reduced oxide nanoparticles. **a**, Pristine ZnO. **b**, 5% Li treated ZnO. **c**, Pristine SnO_2. **d**, 2% Li treated SnO_2. **e**, Pristine CeO_2. **f**, 5% Li treated CeO_2. The particle size of the oxide nanoparticles keeps almost the same before and after lithium reduction, indicating grain growth did not occurred in the grinding process. Scale bars, 100 nm.

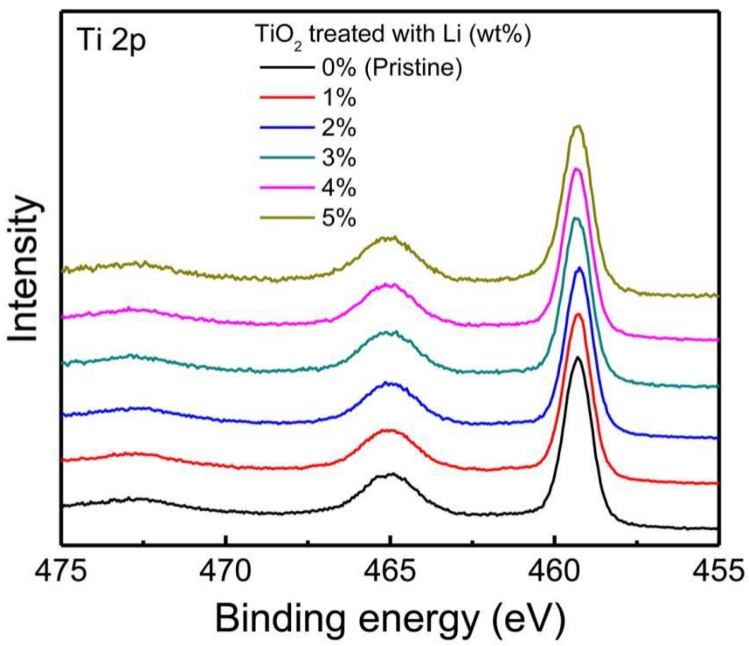

Supplementary Figure 6 | XPS spectra (Ti 2p) of pristine and lithium reduced TiO_2 nanoparticles. The binding energy of the TiO_2 nanoparticles keeps almost the same and no obvious binding energy shift can be found.

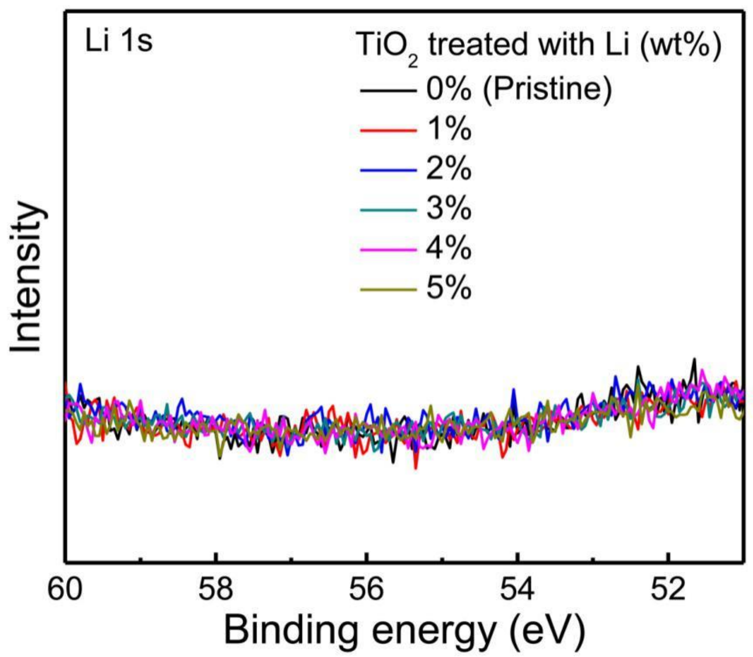

Supplementary Figure 7 | XPS spectra (Li 1s) of pristine and lithium reduced TiO_2 nanoparticles. It is apparent that no peaks correspond to Li (55.5 eV) can be found in the samples, means the generated lithium oxides have been dissolved and removed in the washing process.

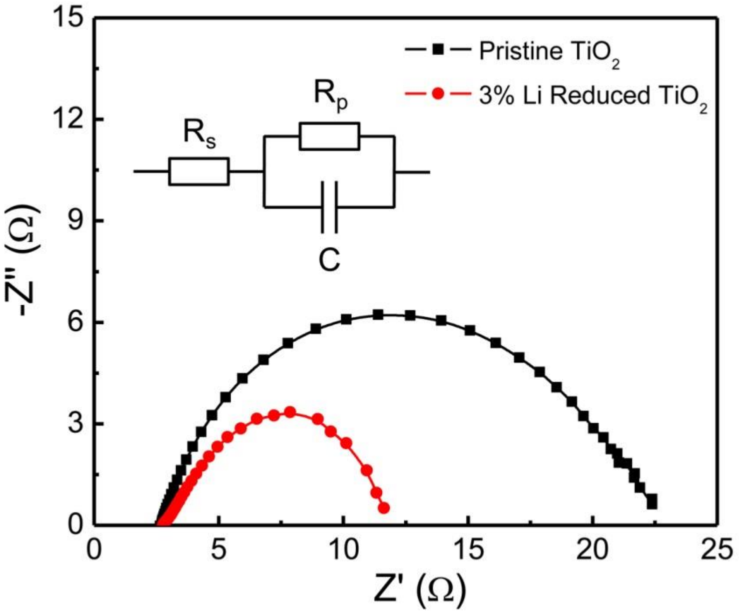

**Supplementary Figure 8 | ** The electrochemical impedance spectra of pristine and 3% Li reduced TiO_2 nanoparticles (Inset: equivalent circuit). The resistance decreased from about 20 Ω to 9 Ω after lithium reduction treatment, suggesting the significantly increased conductivity of TiO_2 nanoparticles due to the implanted defects.

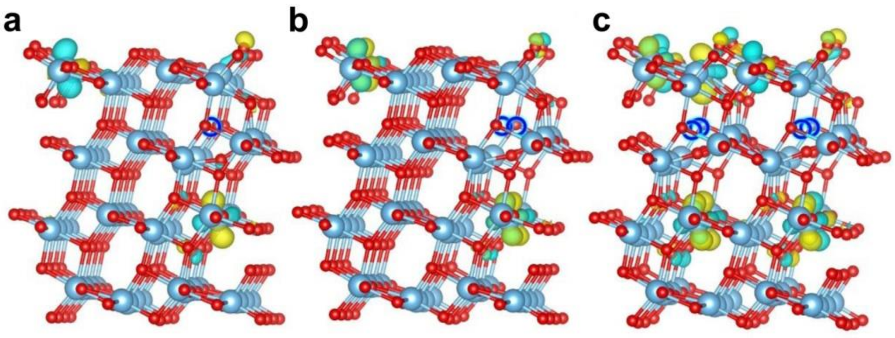

Supplementary Figure 9 | ** The side view of defect TiO_2(101) with different subsurface O_v (blue circles) concentrations. Here, the 1O_v (a**), 2O_v (**b**) and 4O_v (**c**) per formula were considered in our simulations, and the O_v stay in the subsurface. The spin density of the occupied states for different O_v concentrations is also shown in yellow and blue contours for the two different phases. The red and light blue spheres represent O and Ti atoms, respectively. O_vs are shown in the blue circles.

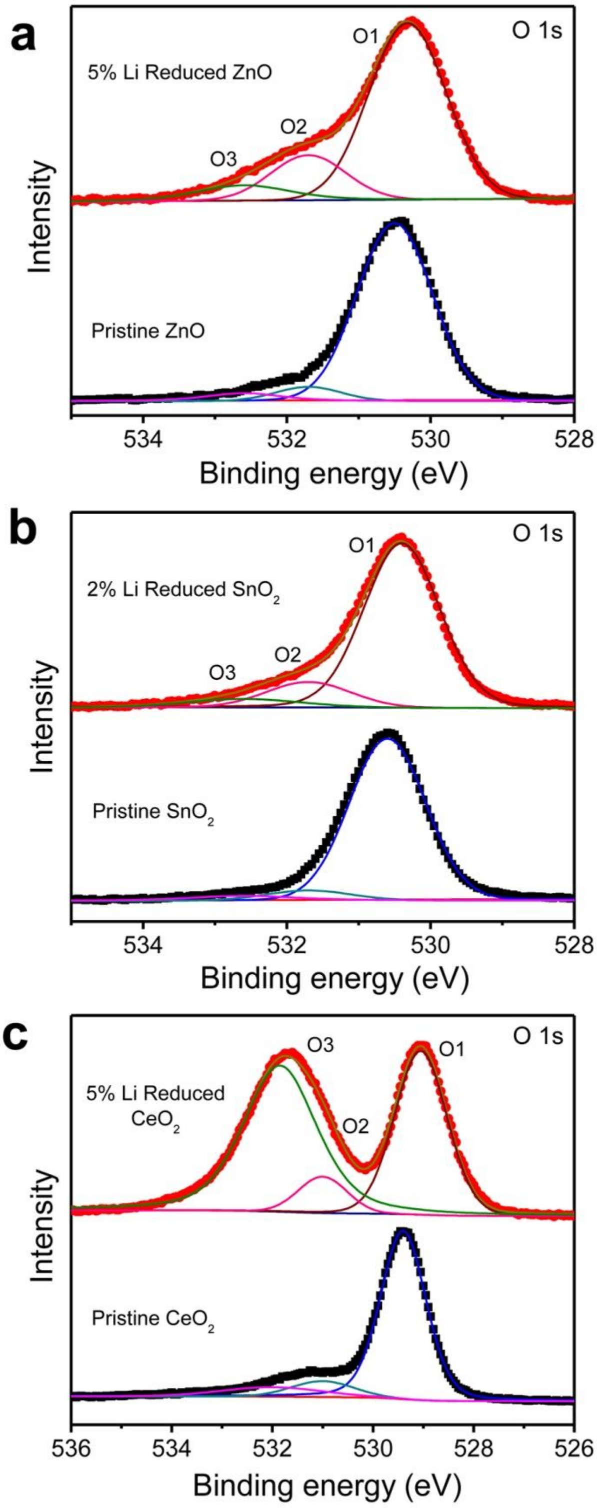

Supplementary Figure 10 | XPS spectra (O1s) of pristine and lithium reduced oxide nanoparticles. **a**, ZnO. **b**, SnO_2. **c**, CeO_2. The three peaks labeled as O1, O2 and O3 can be attributed to the lattice oxygen, oxygen defects and surface adsorbed oxygen species, respectively. It is obvious that all of the oxide nanoparticles demonstrate significantly increased oxygen defects after lithium reduction treatment.

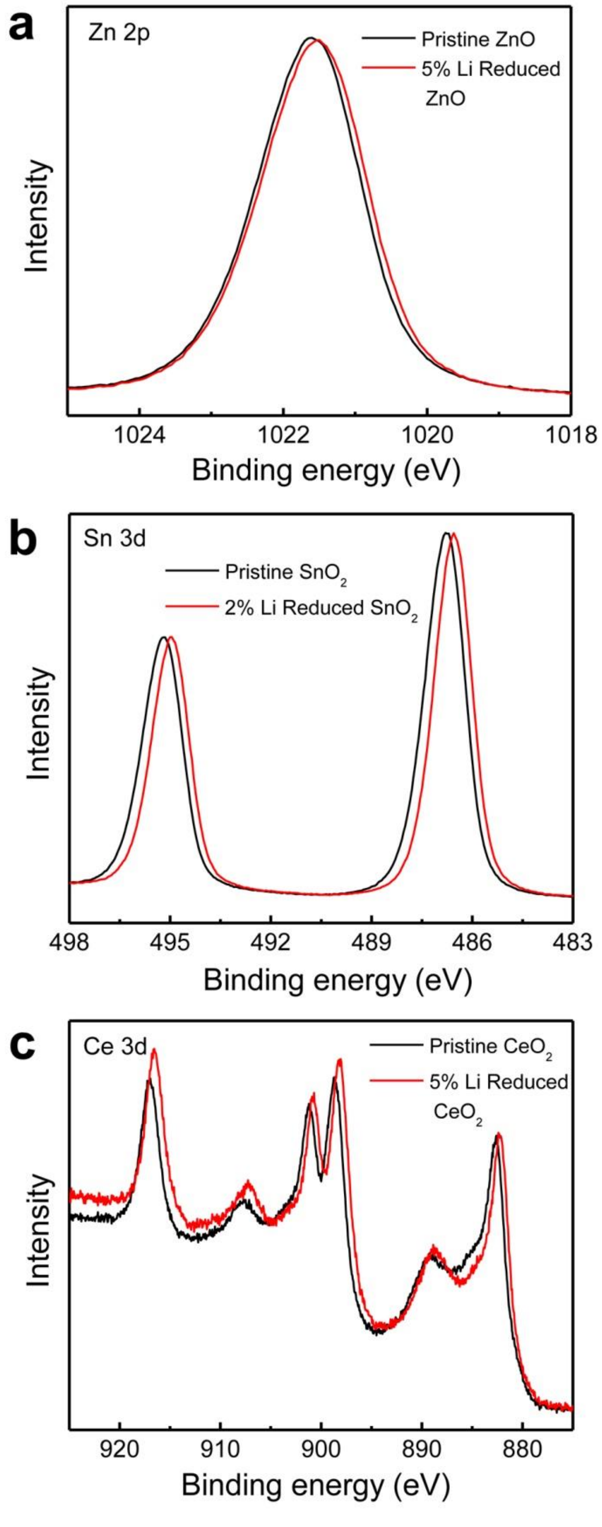

Supplementary Figure 11 | XPS spectra of pristine and lithium reduced oxide nanoparticles. **a**, Zn 2p of ZnO. **b**, Sn 3d of SnO_2. **c**, Ce 3d of CeO_2. It is clear that the binding energy of the metal ions decreased after lithium reduction due to the introduction of defects.

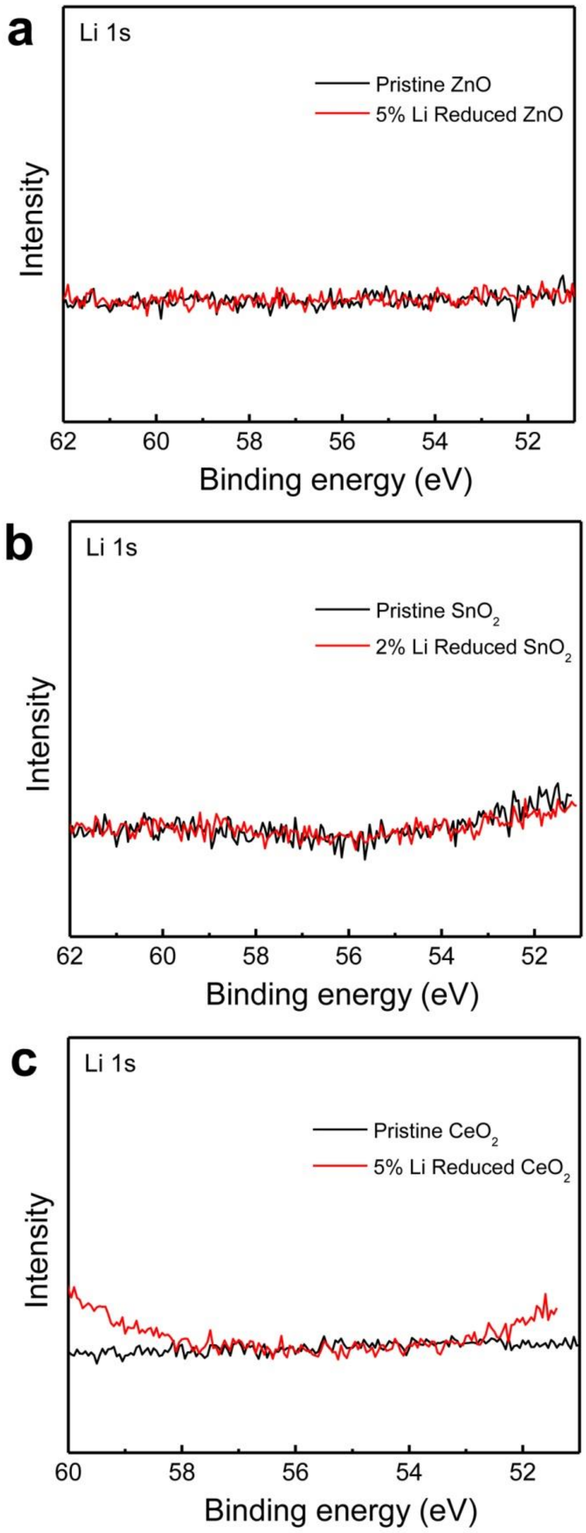

Supplementary Figure 12 | ** XPS spectra (Li1s) of pristine and lithium reduced oxide nanoparticles. **a, ZnO. **b**, SnO_2. **c**, CeO_2. No peaks correspond to Li (55.5 eV) can be found in the oxides, indicating the generated lithium oxides have been completely dissolved and removed in the washing process.

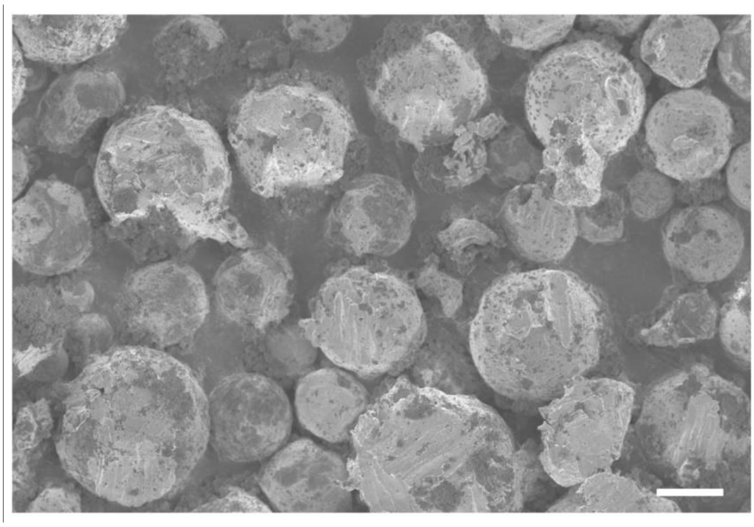

**Supplementary Figure 13 | ** SEM micrograph of lithium powders. Scale bar, 20 μm.

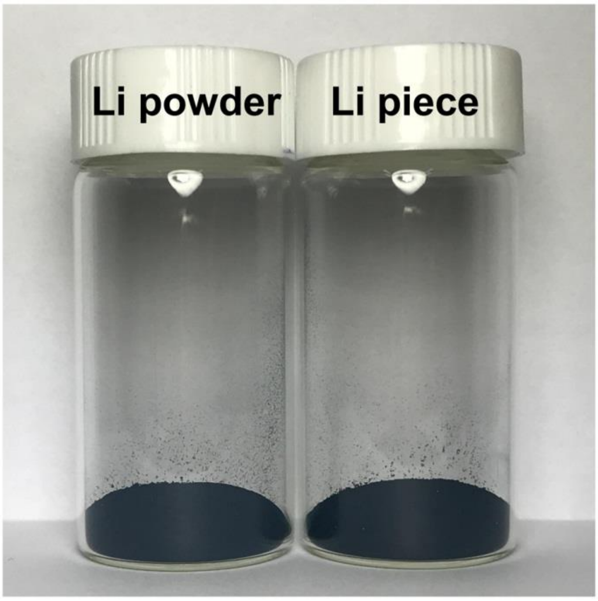

Supplementary Figure 14 | Photograph of TiO_2 nanopowders reduced by 5 wt% Li powders and Li piece respectively.

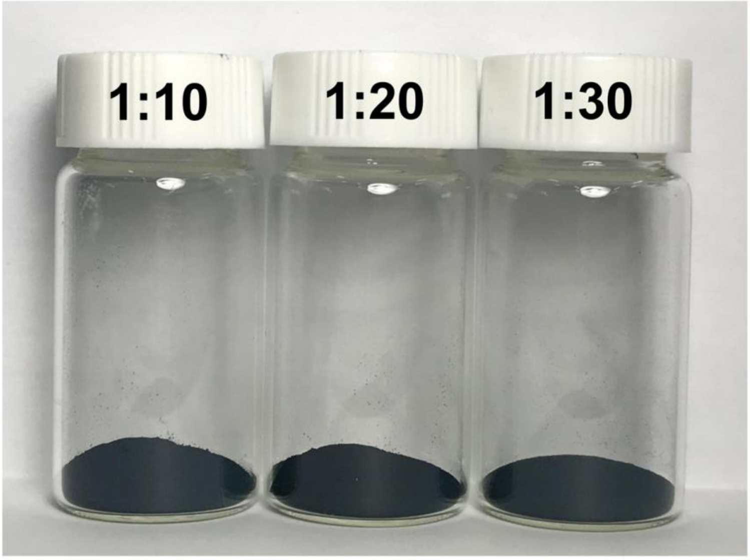

Supplementary Figure 15 | Photograph of TiO_2 nanopowders reduced by 5 wt% Li powders with different weight ratio of oxides to DMC.

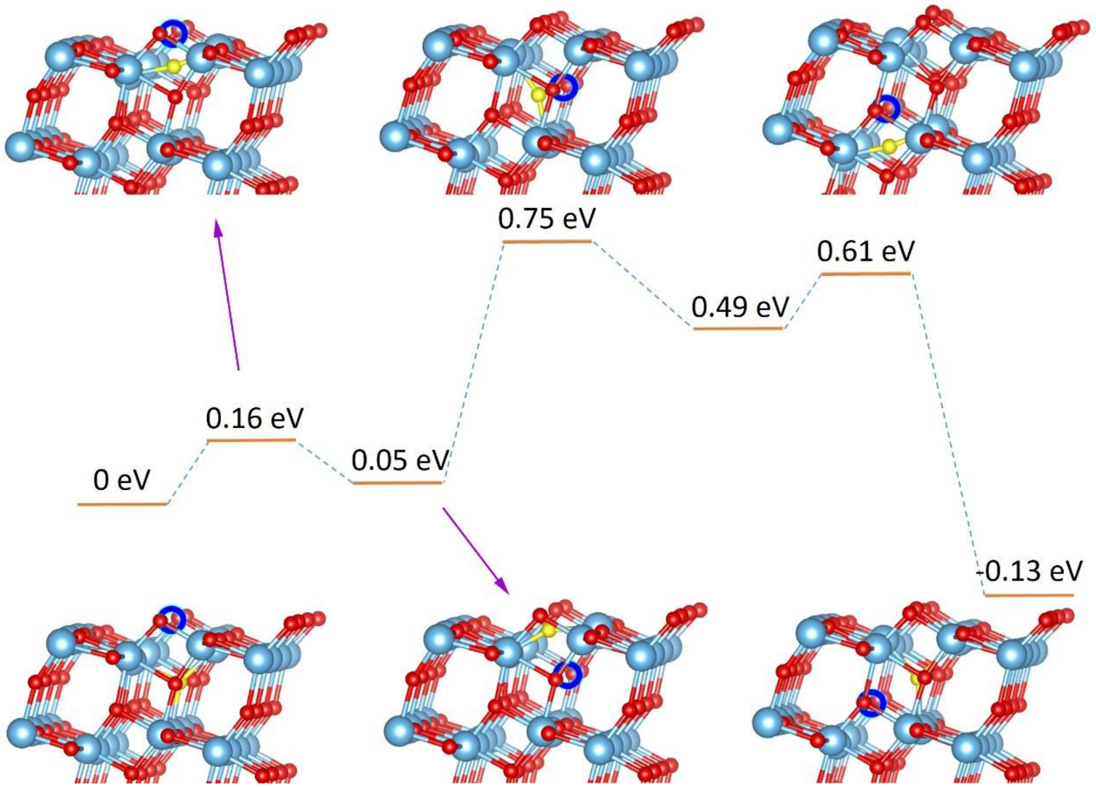

Supplementary Figure 16 | The reaction pathway of the O_v diffusion from surface (the left one) to subsurface (the right one). The total energy of surface O_v is set as the zero energy for reference. The light blue and red balls are Ti atoms and O atoms, respectively. The yellow ball stands for the moving oxygen atom during the O_v diffusion. The blue circle denotes the O_v defect. The transition states are shown on the up panel and the stable O_v sites are shown on the down panel.

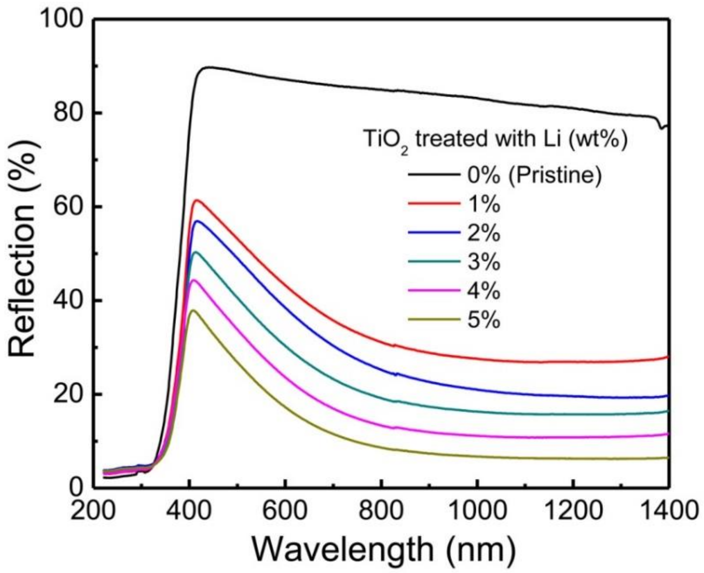

**Supplementary Figure 17 | ** UV–Vis-NIR diffuse reflectance spectra of pristine and lithium reduced TiO_2 nanoparticles. In the visible and NIR region, the TiO_2 nanoparticles demonstrate significantly increased optical absorption, in which the optical absorption increase with the increase of the added lithium content due to the increased defects.

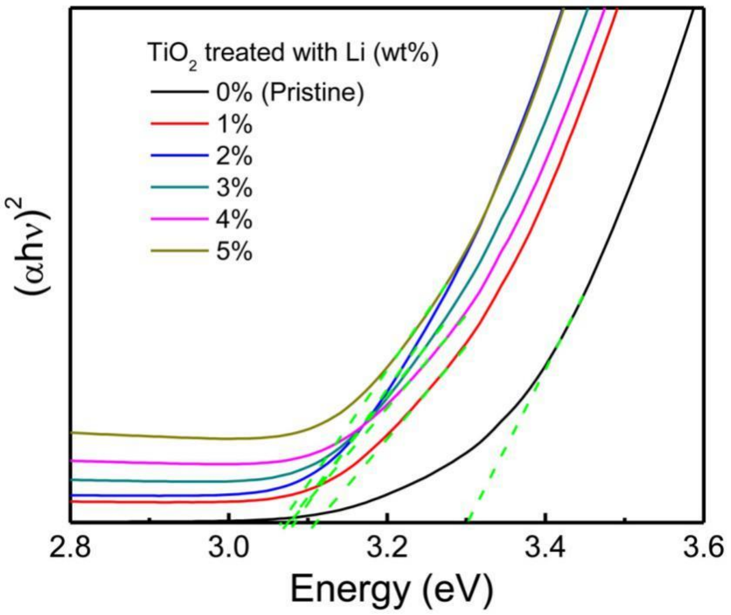

**Supplementary Figure 18 | ** Tauc plots of pristine and lithium reduced TiO_2 nanoparticles. The bandgap was fitted according to the Tauc plot. It shows that the bandgap of pristine TiO_2 is 3.3 eV and that of lithium reduced TiO_2 is about 3.1 eV, suggesting the bandgap has been narrowed about 0.2 eV due to the introduced defects.

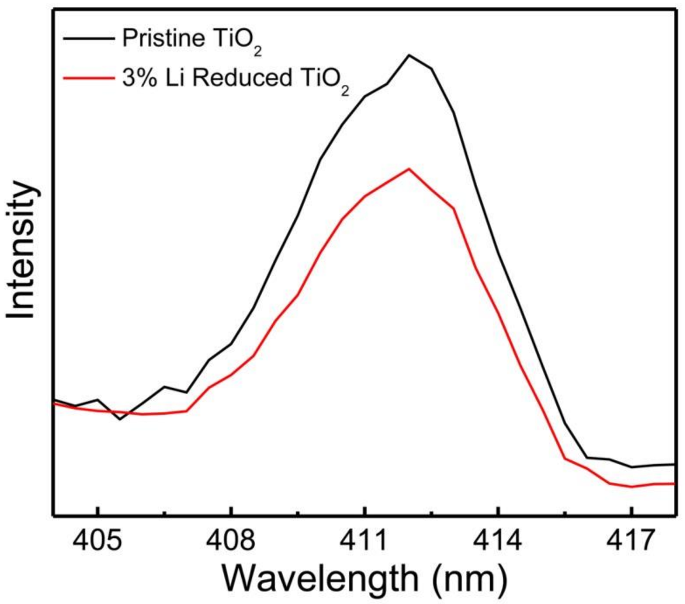

Supplementary Figure 19 | Solid steady state fluorescence spectra of pristine and lithium reduced TiO_2 nanoparticles. The lower fluorescence emission intensity of defective TiO_2 implies that its recombination of excited state electron hole-pairs is less serious than that of pristine TiO_2, indicating defective TiO_2 with longer carriers' lifetime.

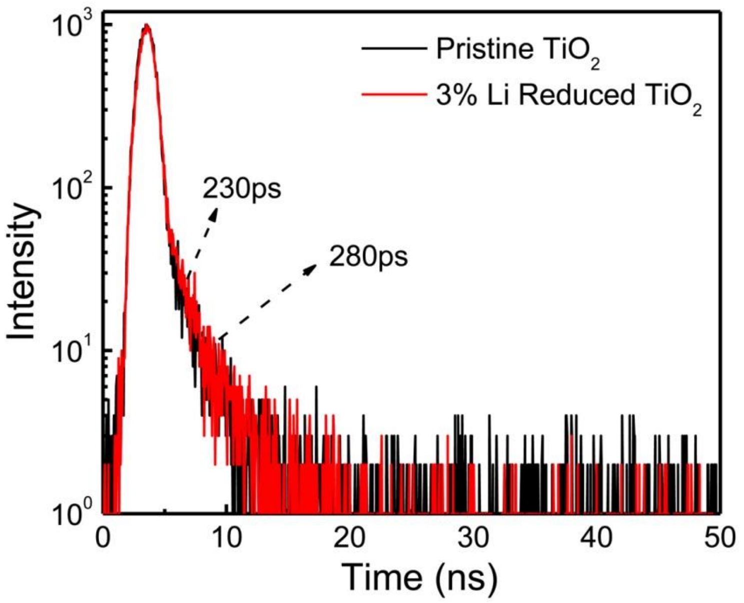

Supplementary Figure 20 | Transient state fluorescence spectra of pristine and lithium reduced TiO_2 nanoparticles. The results showed that the lifetime of photogenerated carriers in defective TiO_2 (280ps) is longer than that of pristine TiO_2 (230ps), suggesting the defects promote the separation of photogenerated carriers indeed.

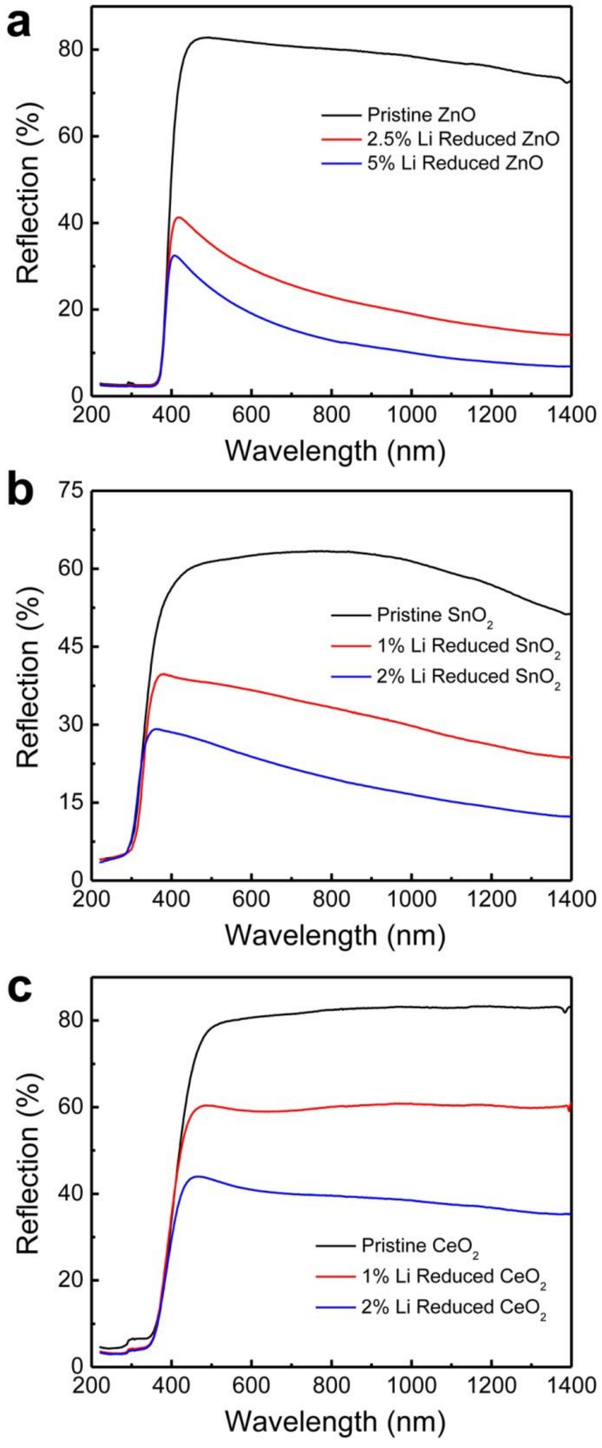

Supplementary Figure 21 | UV-Vis-NIR diffuse reflectance spectra of pristine and lithium reduced oxide nanoparticles. **a**, ZnO. **b**, SnO_2. **c**, CeO_2. It is apparent the oxide nanoparticles demonstrate significantly increased optical absorption in visible and near infrared light region after lithium reduction due to the introduced defects.

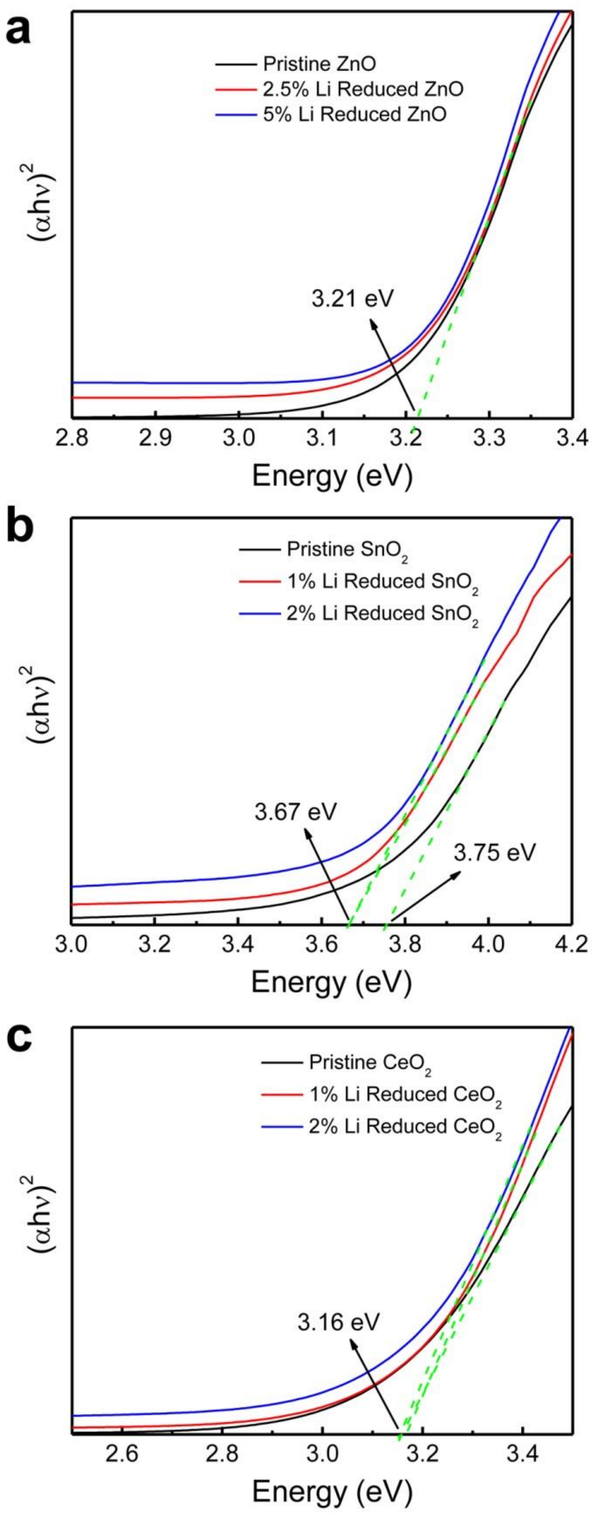

Supplementary Figure 22 | Tauc plots of pristine and lithium reduced oxide nanoparticles. **a**, ZnO. **b**, SnO_2. **c**, CeO_2. The fitted bandgap of the oxides were marked and we can see that they keep almost unchanged after lithium reduction.

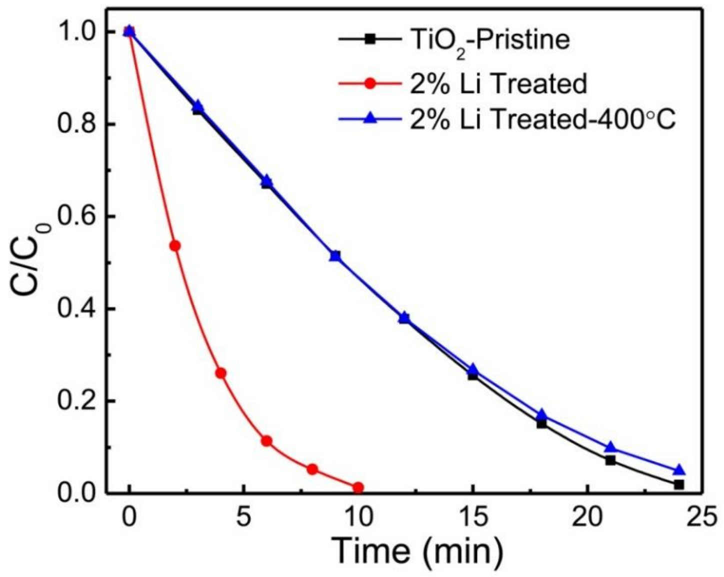

**Supplementary Figure 23 | ** Photocatalytic degradation of RhB by pristine TiO_2, 2% Li treated TiO_2 before and after annealing. The lithium reduced TiO_2 revealed obviously declined photocatalytic activity after annealing and almost the same with pristine TiO_2, indicating the implanted defects in TiO_2 is the main factor for the improved photocatalytic activity.

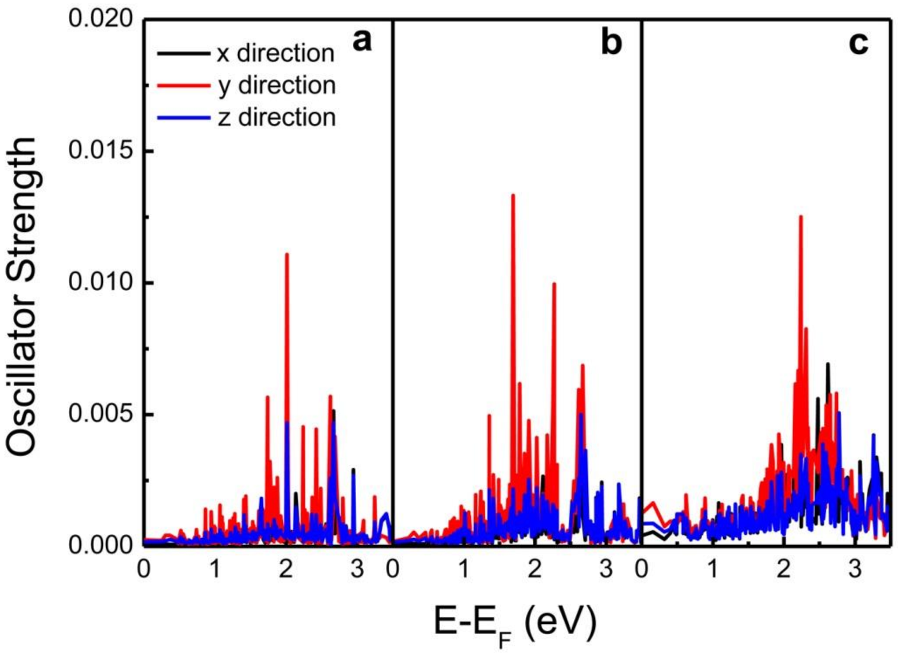

Supplementary Figure 24 | The oscillator strength of reduced TiO_2 surface with $1O_v$ (**a**), $2O_v$ (**b**) and $4O_v$ (**c**). And the strength was calculated for transitions from gap states to conduction band. x, y, z direction represent three different directions of transition dipole moment.

Supplementary Tables

Supplementary Table 1. The enthalpy change ΔH for lithium oxidation using different metal oxide (MO)

MO	TiO_2	SnO_2	ZnO
ΔH (eV)	-4.03	-3.66	-5.05

Supplementary Table 2. Comparison of photocatalytic hydrogen evolution activity with the available experiments

Catalyst	Hydrogen evolution rate (mmol g^{-1} h^{-1})	Light source	SoA	Refs
1% Au/TiO_2	10.2	Iron halogenide mercury arc lamp, 250 W	6% CH_3OH	1
0.6% Pt/TiO_2	10	AM 1.5 solar simulator	50% CH_3OH	2
1% PtO/TiO_2	4.4	Xe lamp, 300 W	30% CH_3OH	3
1% Pt/TiO_2	29	Xe lamp, 300 W	50% CH_3OH	4
1% Pt/TiO_2	6.32	Xe arc lamp, 300 W	20% CH_3OH	5
1% Pt/TiO_2	6.5	Xe lamp, 300 W	25% CH_3OH	6
1% Pt/TiO_2	2.15	Xe arc lamp, 300 W	20% CH_3OH	7
0.5% Pt/TiO_2	6.4	Hg lamp, 300 W	25% CH_3OH	8
1% Pt/TiO_2	43.2	Xe lamp, 400 W	20% CH_3OH	9
1% Pt/TiO_2	4	Xe lamp, 200 W	10% CH_3OH	10
0.5% Pt/TiO_2	15	Xe lamp, 300 W	20% CH_3OH	11
1% Pt/TiO_2	2	Xe lamp, 300 W	30% CH_3OH	12
0.57% Pd/TiO_2	3.32	Xe lamp, 300 W	20% CH_3OH	13
1% Pt/TiO_2	2.41	Xe lamp, 300 W	20% CH_3OH	14
0.5% Pt/TiO_2	5.2	Xe lamp, 300 W	20% CH_3OH	15
1% Pt/TiO_2	10.6	Xe lamp, 300 W	50% CH_3OH	16
0.5% Pt/TiO_2	1.5	Xe lamp, 300 W	20% CH_3OH	17
1% Pt/TiO_2	41.8	Xe lamp, 300 W	20% CH_3OH	This work

Supplementary Reference:

1. Chiarello, G. L., Forni, L. & Selli, E. Photocatalytic hydrogen production by liquid- and gas-phase reforming of CH_3OH over flame-made TiO_2 and Au/TiO_2. *Catal. Today* **144**, 69-74 (2009).

2. Chen, X. B., Liu, L., Yu, P. Y. & Mao, S. S. Increasing solar absorption for photocatalysis with black hydrogenated titanium dioxide nanocrystals. *Science* **331**, 746-750 (2011).

3. Li, Y. H. *et al.* Unidirectional suppression of hydrogen oxidation on oxidized platinum clusters. *Nat. Commun.* **4**, 2500 (2013).

4. Cai, J. M. *et al.* In situ formation of disorder-engineered TiO_2(B)-anatase heterophase junction for enhanced photocatalytic hydrogen evolution. *ACS Appl. Mater. Interfaces* **7**, 24987-24992 (2015).

5. Tian, J., Leng, Y. H., Cui, H. Z. & Liu, H. Hydrogenated TiO_2 nanobelts as highly efficient photocatalyticorganic dye degradation and hydrogen evolution photocatalyst. *J. Hazard. Mater.* **299**, 165-173 (2015).

6. Tan, H. Q. *et al.* A facile and versatile method for preparation of colored TiO_2 with enhanced solar-driven photocatalytic activity. *Nanoscale* **6**, 10216-10223 (2014).

7. Zheng, Z. K. *et al.* Hydrogenated titania: Synergy of surface modification and morphology improvement for enhanced photocatalytic activity. *Chem. Commun.* **48**, 5733-5735 (2012).

8. Wang, Z. *et al.* Visible-light photocatalytic, solar thermal and photoelectrochemical properties of aluminium-reduced black titania. *Energy Environ. Sci.* **6**, 3007-3014 (2013).

9. Sinhamahapatra, A., Jeon, J. P. & Yu, J. S. A new approach to prepare highly active and stable black titania for visible light-assisted hydrogen production. *Energy Environ. Sci.* **8**, 3539-3544 (2015).

10. Li, L. *et al.* Sub-10 nm rutile titanium dioxide nanoparticles for efficient visible-light-driven photocatalytic hydrogen production. *Nat. Commun.* **6**, 5881 (2015).

11. Lin, T. Q. *et al.* Effective nonmetal incorporation in black titania with enhanced solar energy utilization. *Energy Environ. Sci.* **7**, 967-972 (2014).

12. Zhao, Z. *et al.* Effect of defects on photocatalytic activity of rutile TiO2 nanorods. *Nano Res.***12**, 4061-4071 (2015).

13. Xu, Y. F. *et al.* Pd-catalyzed instant hydrogenation of TiO_2 with enhanced photocatalytic performance. *Energy Environ. Sci.* **9**, 2410-2417 (2016).

14. Hu, W. Y. *et al.* Facile strategy for controllable synthesis of stable mesoporous black TiO_2 hollow spheres with efficient solar-driven photocatalytic hydrogen evolution. *J. Mater. Chem. A* **4**, 7495-7502 (2016).

15. Zhu, G. L. *et al.* Black titania for superior photocatalytic hydrogen production and photoelectrochemical water splitting. *ChemCatChem* **7**, 2614-2619 (2015).

16. Wang, Y. T. *et al.* Hydrogenated cagelike titania hollow spherical photocatalysts for hydrogen evolution under simulated solar light irradiation. *ACS Appl. Mater. Inter.* **8**, 23006-23014 (2016).

17. Zhang, K. F. *et al.* Black N/H-TiO_2 nanoplates with a flower-like hierarchical architecture for photocatalytic hydrogen evolution. *ChemSusChem* **9**, 2841-2848 (2016).
